# An Interactive Control Algorithm Used for Equilateral Triangle Formation with Robotic Sensors

**DOI:** 10.3390/s140407229

**Published:** 2014-04-22

**Authors:** Xiang Li, Hongcai Chen

**Affiliations:** Department of Physics and Electronic Engineering, Hanshan Normal University, Chaozhou 521041, China; E-Mail: chc2523136@hstc.edu.cn

**Keywords:** robotic sensor, interactive control, stability analysis, computer simulation

## Abstract

This paper describes an interactive control algorithm, called Triangle Formation Algorithm (TFA), used for three neighboring robotic sensors which are distributed randomly to self-organize into and equilateral triangle (E) formation. The algorithm is proposed based on the triangular geometry and considering the actual sensors used in robotics. In particular, the stability of the TFA, which can be executed by robotic sensors independently and asynchronously for E formation, is analyzed in details based on Lyapunov stability theory. Computer simulations are carried out for verifying the effectiveness of the TFA. The analytical results and simulation studies indicate that three neighboring robots employing conventional sensors can self-organize into E formations successfully regardless of their initial distribution using the same TFAs.

## 1. Introduction

There is a growing research interest in formation control theories for multi agent systems. Multi-agent systems are typically applied to tasks that cannot be handled by individual agents [1]. The main inspiration of the research in this field stems from the collaborative behaviors commonly observed in social animals, for instance, flocks of birds, and schools of fishes [2]. However, there are only local interactive rules utilized behind these complex behaviors [3,4]. Formation control of a multi-agent system has potential applications in many other domains other than the robotics such as sensor networks [5], surveillance missions [6], and search and rescue [7].

The pioneering formation model, flocking, was created successfully and applied for computer graphics by Reynold [8]. A main contribution of Reynold's work is that it demonstrates that a global behavior can emerge from local interactive rules used by the agents. Balch and Arkin [9] proposed a behavior-based control approach for a small group of mobile robots (up to four) to strive to maintain some specific geometric formations. Formation task of a robot is decomposed into some basic behaviors and the aim of motion control can be gained through synthesizing these basic behaviors. The robots are heterogeneous since each robot's position in the formation depends on an ID number. Subsequently, Balch and Hybinette [10,11] extended the behavior-based approach to large scale robot formations. The behavior-based approach doesn't benefit stability analysis of formation.

In this study, we are concerned with three homogeneous robots employing commonly available sensors to group into an equilateral traingle (E) formation based on triangular geometry. Reif and Wang [12] extended the potential field approach which is widely applied to navigation of single robots to control multiple robots in formations for the first time. In their work, local minima had to be treated and potential function value would tend to reach infinity when two robots are close enough, which isn't realizable in practice. Kim *et al.* [13] presented a set of analytical guidelines for designing potential functions to avoid local minima for a number of representative scenarios. An important issue that has to be addressed is the selection of proportional parameters representing the relative strength of attractive and repulsive forces in a complexity and uncertainty environment. For these potential field approaches, the regular even local formation is not taken into account in formation control. Spears *et al.* [14,15] proposed an artificial physics-based framework for controlling a group of robots using attractive/repulsive forces between them. The decision of each robot depends on the local information. However, this approach tends to make the robots cluster unpredictably and also requires that robots must be close enough to each other at the start. To circumvent these problems, inspired by physics, a decentralized control mechanism based on virtual spring mesh was developed by Shucker and Bennett [16,17] for the deployment of robotic macrosensors. Each robotic sensor in the macrosensor interacts with its neighbors by using the physics model of virtual spring mesh abstraction while the neighbors are required to satisfy the acute condition. Related model parameters have to be set carefully in practice. Chen and Fang [18,19] introduced a geometry-based control approach for multi-agent aggregations while collision avoidance between members still uses a potential function-based method. The value of a potential function exerting on an agent tends to reach infinity when it is close enough to its neighbors and regular formation isn't involved there. Lee *et al.* [20,21] described a geometric motion planning framework which is constructed upon a geometric method for a group of robots in formation. The assumption of three neighboring robots starting from an acute triangle configuration is their major weakness though local regular triangular formation is considered in their work. However, in our study a group of three robots with basic sensor units that are capable of detecting each other are desired to organize into a basic E formation starting from any arbitrary initial distribution.

The modeling and stability analysis of the basic system considered in our study can also be extended and considered as a large interconnected system. Recently, analysis and stabilization of multiple time-delay interconnected systems is also receiving increasing attention from the scientific community [22–26]. In practice, the interconnected systems include electric power systems, process control systems, different types of societal systems, and so on. Chen and Chiang [25] extended the T-S fuzzy control representation to the stability analysis for nonlinear interconnected systems with multiple time-delays using LMI theory and proposed a LMI-based stability criterion which can be solved numerically. In [26], a fuzzy robust control design which combines H infinity control performance with T-S fuzzy control for the control of delayed nonlinear structural systems under external excitations is presented by them. The modeling error is further considered for resolution in this work. The emphasis of the work of Chen and Chiang is on the stability and stabilization of complex interconnected systems which are usually modeled as a unified formula. In contrast to many systems considered in the literature, in our study we are concerned with local interactive rule design so that an effective global behavior emerges from these rules. By comparison, the time-delay and modeling error are not immensely significant in our study. Hence, they are not taken into account in the design and stability analysis of the TFA.

The remainder of this paper is organized as follows: Section 2 gives the problem statement including the state transition model and motion control equation of robotic sensors. The detailed design procedure of the interactive control algorithm, TFA, is presented in Section 3. In Section 4, we conduct the stability analysis of the TFA which can be executed by robotic sensors independently and asynchronously for E formation. Section 5 demonstrates E formation behavior and typical formation convergences of three neighboring robotic sensors through computer simulations. Finally, conclusions and future work are stated in Section 6.

## 2. Problem Statement

We consider low cost homogeneous robots embodying simple and commonly available sensors. This means that the members cannot have strong capability, e.g., remote communication, and can only interact with neighbors or their environment. In fact, compared with single robotic sensors with complex structure and comprehensive function, there is less probability for a simple robotic sensor to be destroyed while performing tasks. In the following subsections, the state transition model and motion control equation of a robotic sensor, and definitions involved later are stated first.

### 2.1. State Transition Model

In our model, we assume that a real robotic sensor consists of four basic hardware components: (1) proximity sensor (such as infra-red sensor, sonar sensor, camera, *etc.*), to detect the distances between itself and neighbors; (2) digital compass, to detect the azimuths of neighbors within its local coordinate system; (3) central processor, to compute goal position using the designed interactive control algorithm based on the local information gained by (1) and (2); (4) actuator, to drive the robot and its sensor unit to move with the velocity calculated by motion control equation based on the goal position. The first two components together complete the collection of the local information.

**Figure 1.**
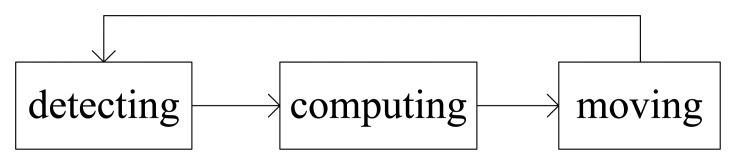
The state transition model of a robotic sensor.

To emphasize the development of a cooperative mechanism, the model abstract of a robotic sensor is assumed in following statement, where hardware composition and action characteristics of robotic sensors are taken into account:

*Assumption 1*: A robotic sensor only has the ability of gaining local information and possesses three executable states: detecting, computing and moving. Transition happens between these three states subsequently and periodically when the robotic sensor runs, as shown in Figure 1, and there is no time-delay and disturbance during the transitions.

In fact, execution of each state adds to the time cost and the transition between any two states also has a time-delay for robotic sensors. During the detection stage, a robotic sensor needs time to detect the distances from its neighbors and simultaneously determine the azimuths of corresponding neighbors, especially for the case that the robotic sensor which is equipped with only one infra-red sensor and has to perform 360° rotation to collect information. The calculation of goal position is based on the local information and it also adds to the time cost.

**Figure 2.**
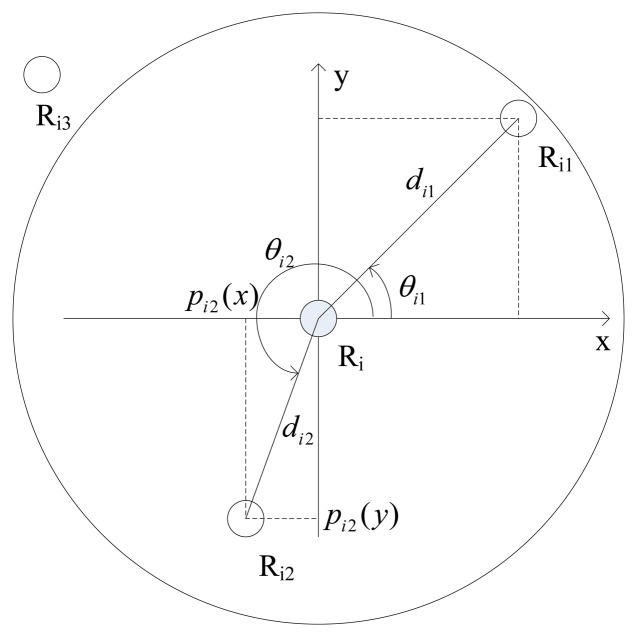
The detection of neighbor's local position information

For the robotic sensor R_i_, the details of goal position calculation using detected distances and azimuths of its two neighbors is described in Figure 2. There are four robotic sensors which are distributed randomly in the plane, while only robotic sensors R_i1_ and R_i2_ are located within the detectable radius r_s_ of the robotic sensor R_i_, and robotic sensor R_i3_ is not detectable by R_i_ since R_i3_ is located outside the detectable radius r_s_ of R_i_. *d_i_*_1_ and *d_i_*_2_denote the distances of R_i1_ and R_i2_ from R_i_ respectively, while *θ_i_*_1_ and *θ_i_*_2_ denote the local azimuths of R_i1_ and R_i2_ respectively within R_i_'s local coordinate system. Consequently, R_i_ is able to calculate R_i1_'s local position *p_i_*_1_(*p_i_*_1_(*x*), *p_i_*_1_(*y*)) according to Equation (1) based on the detected local information about R_i1_. Similarly, the local position of R_i2_ can also be calculated. However, R_i_ can't detect any position information about R_i3_ since R_i3_ is not detectable to R_i_:

(1) $$\{\begin{array}{ll} p_{i1}(x) & =d_{i1}\cos\theta_{i1}\\ p_{i1}(y) & =d_{i1}\sin\theta_{i1} \end{array}$$

The condition for three robotic sensors to configure an E formation is that each robot-sensor unit has to be positioned within the detection ranges of the other two. For the rest of this paper, unless stated otherwise this condition is assumed to be satisfied when they are distributed at the start. Robotic sensor R_i_ collects the local position information of its other two neighbors during the detection stage, then executes an interactive control algorithm, TFA, to calculate its goal position, and finally puts the goal position into a motion control equation to output the desired motion state. The motion state means goal velocity which has two properties of speed and direction. The actuator of R_i_ will drive R_i_ to move with the velocity.

Among three robotic sensors, any one robotic sensor and its position are denoted by R_i_ and *p_i_* respectively, and the other two may be denoted by R_i1_, R_i2_ and accordingly their positions by *p_i_*_1_, *p_i_*_2_. *NP_i_* denotes the position set {*p_i_*_1_, *p_i_*_2_} of R_i_'s neighbors *d_j,k_* denotes the distance between *p_j_* and *p_k_*. _ID_ denotes the index set {*i*, *i*1, *i*2} of the robotic sensors.

*Definition 1* (Generalized triangle, G): Generalized triangle, G, is defined as an arbitrary three-point formation determined by position set, G = {*p*_1_, *p_i_*_1_, *p_i_*_2_}, where three elements may be collinear.
*Definition 2* (Equilateral triangle, E): Equilateral triangle, E, is defined as the G where the distances between any pair in{*p*_1_, *p_i_*_1_, *p_i_*_2_} are equal to D, where D is called side-length of E.

Based on the definitions of G and E, E formation behavior of three neighboring robotic sensors may be stated as follows:

*Problem 1* (E formation behavior): Three neighboring robotic sensors self-organize into the desired E formation with side-length D through cooperation between each other starting from arbitrary initial G formation which depends on their initial distribution.

### 2.2. Motion Control Equation

At each time step, robotic sensor R_i_ uses collected local information to calculate its goal position. This goal position is considered invariable during the interval between two time steps. Therefore, we introduce following motion control equation for R_i_:

(2) $${\dot{p}}_{i}=-\text{C}(p_{i}-p_{ig})$$

where *p_i_* and *p_ig_* denote the current and goal positions of R_i_ respectively; C is a constant relying on the maximum speed ‖*v*_max_‖ and detecting radius r of R_i_, which could be determined according to the following procedure. As Seen from Equation (2), during R_i_'s movement, ‖*p_˙i_*‖ = C‖*p_ig_* − *p_i_*‖ holds at all time. For some C, the maximum of the right term, C‖*p_ig_* − *p_ig_*‖, is C r_s_. The left term, ‖*p_˙i_*‖, is the speed of R_i_. As long as ‖*p_˙i_*‖ ≤ ‖ν_max_‖, a proper C could always be found such that ‖*p_˙i_*‖ = C·r*_s_*. Therefore an acceptable C should make Equation (4) satisfied:

(3) $$\text{C}\leq \frac{∥v\max∥}{{\text{r}}_{s}}$$

After selecting a proper value for C, R_i_'s motion state at the next time step is dependent on goal position *p_ig_* as demonstrated in Equation (2). The *p_ig_* is calculated by R_i_ using an interactive control algorithm, TFA, so the design of TFA is key for solving Problem 1.

## 3. Presentation of Interactive Control Algorithm (TFA)

**Figure 3.**
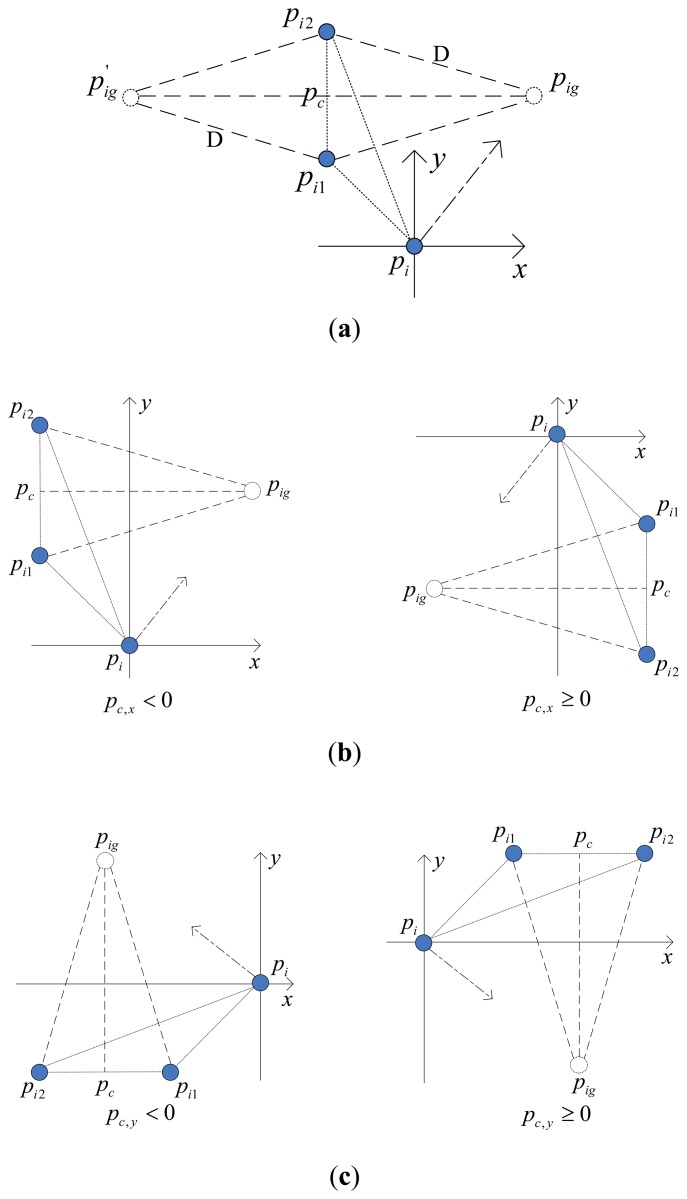

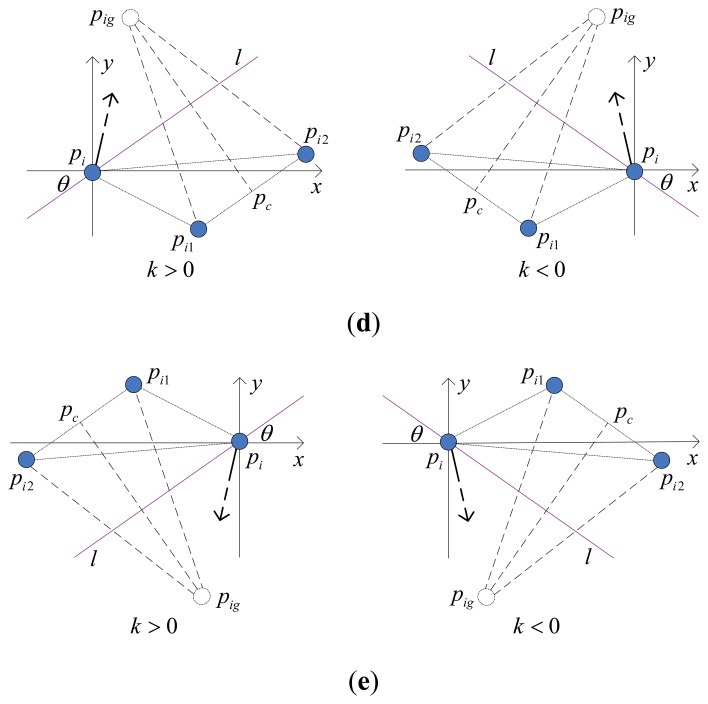
The calculation methods of specific *p_ig_* for R_i_. (**a**) The case where two possible destinations for *p_i_*. (**b**) *p_i_*_1_*p_i_*_2_ is parallel to axis *y*. (**c**) *p_i_*_1_*p_i_*_2_ is parallel to axis *x*. (**d**) *p_c_* under line *l*. (**e**) *p_c_* above (on) line *l*.

During E formation configuration, each robotic sensor executes the same interactive control algorithm and their behaviors are independent and asynchronous. As a member of the group, actions of the member should be beneficial to achieving global task. According to this rule, the goal position for a given robotic sensor R_i_ should be the position from which the distances to the rest of the two neighbors are equal. If this goal position can be calculated then there will be two possible solutions as shown in Figure 3a. This conflict is solved by choosing the one which is closer to R_i_ and denoting it as *p_ig_*. The side-length of desired E formation is denoted by D and D < r_s_ should hold, otherwise the desired E formation will not be possible to achieve as neighbors are not detecting each other. In order to let all robotic sensors have specific goal positions at the same time, the below equation must be satisfied:

(4) $$\begin{array}{lll} d_{j,k}\leq 2D, & \forall j,k\in \text{ID}, & j\neq k \end{array}$$

where *d_j,k_* represents the distance between *p_j_* and *p_k_* which are the positions of robotic sensors R_j_ and R_k_ respectively.

Here, we divide the process of configuring the E formation of three robotic sensors into two stages: adjusting process (APr) and clustering process (CPr). For APr, Equation (4) holds while for CPr it does not. The corresponding algorithms executed on the robotic sensor are called adjusting algorithm (AA) and clustering algorithm (CA), respectively.

### 3.1. Adjusting Process (APr)

During the adjusting process, Equation (4) holds and at the same time each member of the three robotic sensors is able to calculate its specific goal position for the next step. The mathematical significance of *p_ig_* for robotic sensor R_i_ may be stated as Equations (5) and (6) where the sign of ‖ · ‖ means Euler norm-2 which is used for distance solution between any pair of positions in two-dimensional space:

(5) $$P=\{p|∥p-p_{m}∥=D,\forall m\in NP_{i},p\in R^{2}\}$$

(6) $$p_{ig}=\underset{p\in P}{\arg}\left[\min(∥p-p_{i}∥)\right]$$

The calculation method of goal position *p_ig_* of robotic sensor R_i_ under all typical distributions are illustrated within the local coordinate system of R_i_ in Figure 3b–e. In these figures, a hollow circle indicates the goal position and a full circle indicates the robotic sensor position. The arrowhead pointing to *p_ig_* from *p_i_* indicates expected motion direction of R_i_. The center of line segment *p_i_*_1_*p_i_*_2_ is denoted by *p_c_*. The *p_ig_* which has the same distance D from *p_i_*_1_ and *p_i_*_2_ locates on the line which is perpendicular to line *p_i_*_1_*p_i_*_2_ and passes through *p_c_*. *l* represents the line which passes through the origin and is also parallel to line *p_i_*_1_*p_i_*_2_. *k* indicates the slope of line *l* within R_i_'s local coordinate system. *θ* indicates the included angle between line *l* and horizontal axis x. The pseudo code of basic AA algorithm with respect to adjusting process is described in Table 1.

### 3.2. Clustering Process (CPr)

**Figure 4.**
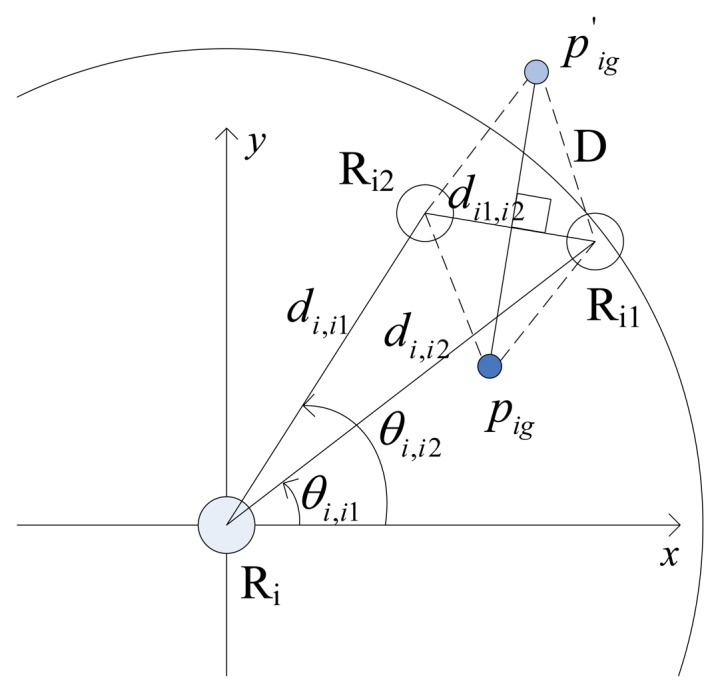
A position distribution and the *p_ig_* of R_i_.

At each time step, robotic sensor R_i_ calculates the distance between the other two neighbors after detection. If this distance satisfies Equation (4), R_i_ can calculate it's the specific *p_ig_* which satisfies Equations (5) and (6) using the AA algorithm. If robotic sensor R_i_ located at *p_ig_*, could form an isosceles triangle together with its two neighbors and the waist lengths are equal to D. R_i_ moving towards *p_ig_* benefits E formation. However, it is not guaranteed that at the same time step the other two neighbors also have specific goal positions, which is completely dependent on their current distribution. A distribution of three robotic sensors, for instance, is shown in Figure 4, where *d_i_*_1_,*_i_*_2_ < D, and the goal position of R_i_ is *p_ig_* (not $p_{ig}^{\prime }$) because *d_ig_*,*_i_*_1_ = *d_ig_*,*_i_*_2_ = D and *p_ig_* is closer to *p_i_* than $p_{ig}^{\prime }$.

Since R_i_ is located so far from R_i1_ that R_i2_ cannot calculate a specific goal position *p_i_*_2_*_g_* to make *d_ig_*,*_i_*_1_ = *d_ig_*,*_i_*_2_ = D hold. After detected the distances *d_i_*,*_i_*_1_ and *d_i_*,*_i_*_2_ using proximity sensor and the azimuths *θ_i_*,*_i_*_1_ and *θ _i_*,*_i_*_2_ using digital compass, R_i_ can calculate the distance between R_i1_ and R_i2_ according to Equation (7):

(7) $$d_{i1,i2}=\sqrt{d_{i,i1}^{2}+d_{i,i2}^{2}-2\cdot d_{i,i1}\cdot d_{i,i2}\cdot \cos(\theta_{i,i2}-\theta_{i,i1})}$$

If Equation (4) doesn't hold, at least one robotic sensor can't calculate a specific goal position because the goal position doesn't exist. They should first cluster together enough prior to the calculation of goal position. Therefore, we define following strategy: when R_i_ found that Equation (4) doesn't hold, it will take the average position of three robotic sensors as its approximate goal position *p_ig_* According to this strategy, when Equation (4) doesn't hold, the three robotic sensors first cluster until Equation (4) is satisfied. After Equation (4) holds, each robotic sensor has its own specific goal position at the same time step and they will join the adjusting process. Adjusting process straightly goes to configure E formation, while clustering process only aims to make three robotic sensors cluster enough to ensure that they all can finally join adjusting process. The pseudo code of basic CA algorithm with respect to clustering process is described in Table 2.

**Figure 5.**
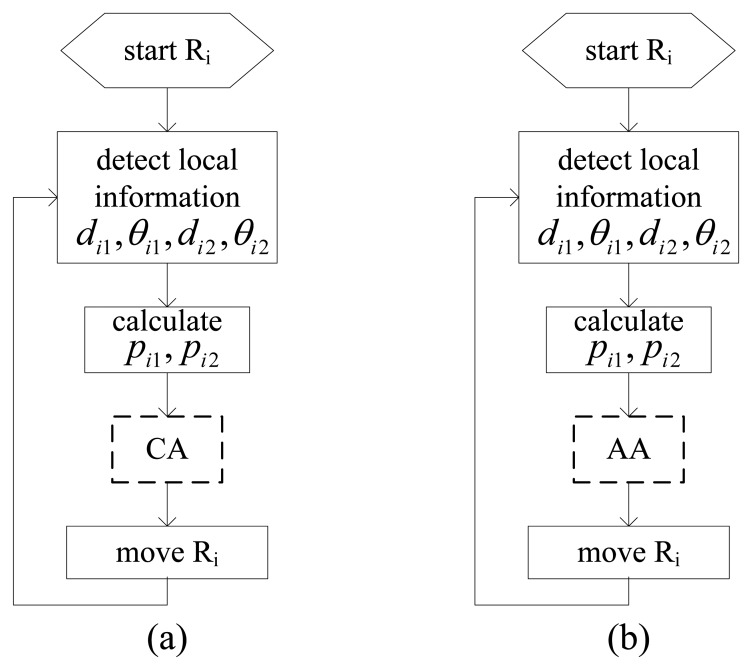
The execution procedures of basic CA and AA algorithms for R_i_.

In fact the CA algorithm itself has no specific requirement for position distribution of three robotic sensors because the approximate goal position, *i.e.*, the average position of them, is available at any given time step. If the three robotic sensors only execute the same basic CA algorithms, they will move towards a common position. The execution procedure of basic CA algorithm for R_i_ is illustrated in Figure 5a.

Different from the CA algorithm, the AA algorithm has the requirement for position distribution of three robotic sensors. In other words, Equation (4) must hold, otherwise the specific goal position will not exist for at least one robotic sensor, which will cause severe problems. The three robotic sensors will go to configure an E formation directly by executing the same basic AA algorithms. The execution procedure of the basic AA algorithm for R_i_ is illustrated in Figure 5b.

### 3.3. TFA Description

A robotic sensor's choice of which process from the clustering and adjusting processes to join is completely dependent on the current distribution of three robotic sensors. If one robotic sensor joins the adjusting process first, it will no longer join the clustering process and go into configuring an E formation directly. If a robotic sensor joins the clustering process first, it will have to join the adjusting process for configuring an E formation after the three robotic sensors cluster enough. The detailed analysis and results on these issues will be presented in the next section. Here, we name the proposed interactive control algorithm Triangle Formation Algorithm (TFA). The same TFA algorithm is executed on each robotic sensor independently and asynchronously while configuring an E formation. For the convenience of description, two definitions are given below for classifying the distributions of three robotic sensors:

*Definition 3* (Clustering Formation, CF): CF is defined as the G formation which doesn't satisfy Equation (4).
*Definition 4* (Adjusting Formation, AF): AF is defined as the G formation which satisfies Equation (4).

**Figure 6.**
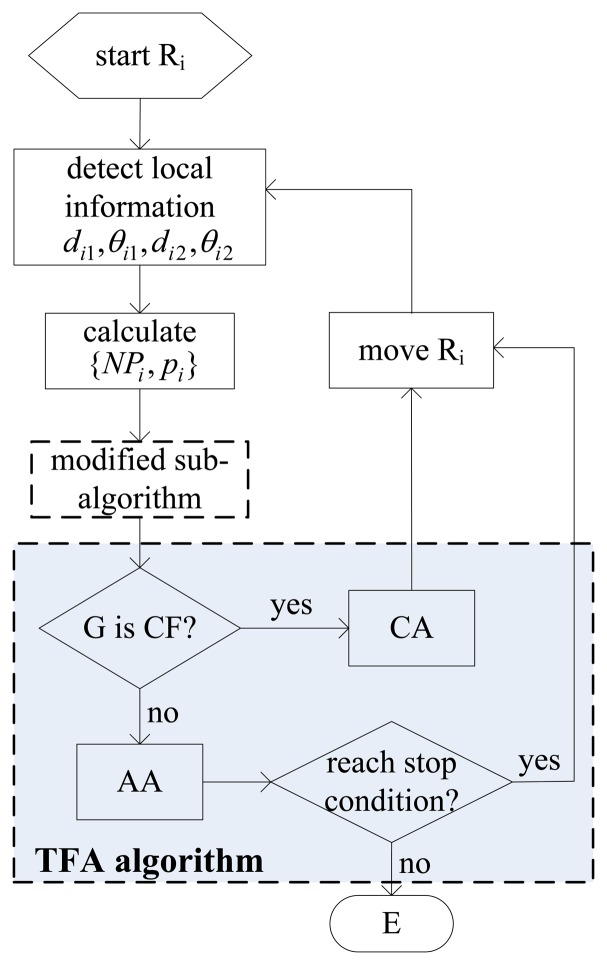
The execution procedure of the TFA algorithm for R_i_.

The G formation, configured by three robotic sensors, only belongs to one type of the CF or AF. The robotic sensor will execute the CA algorithm when it locates in CF, and the AA algorithm in AF. Three robotic sensors can cluster enough through executing the same CA algorithms so as to make Equation (4) hold. Equation (4) needs to be satisfied for the AA algorithm. However these algorithms as parts of the TFA algorithm have the common aim to achieve the E formation. To complete the global task, the AA algorithm is the part each robotic sensor has to execute at the end. For a robotic sensor, the part of the CA algorithm is not guaranteed to be executed. It depends on the current formation configured by the three robotic sensors. Therefore, the TFA algorithm used for three robotic sensors to configure an E formation is composed of the basic CA and AA algorithms. For a robotic sensor to choose which part of the TFA algorithm to execute depends on the type of current formation configured by the three robotic sensors. The pseudo code of the TFA algorithm is described in Table 3. The execution procedure of the TFA algorithm for R_i_ is illustrated in Figure 6.

**Figure 7.**
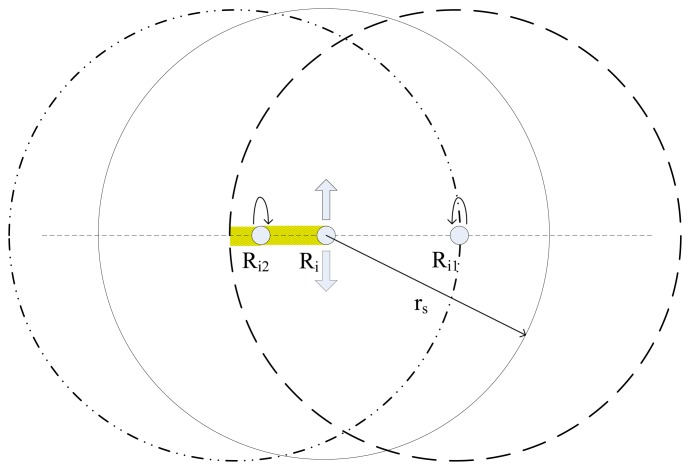
The collinear distribution of three robotic sensors.

In practice when three robotic sensors locate along a line, it may happen that the two robotic sensors at the ends are unable to detect each other due to the robotic sensor located in the middle, but the middle robotic sensor is able to detect the other two neighbors on both sides. Figure 7 indicates this kind of position distribution.

**Figure 8.**
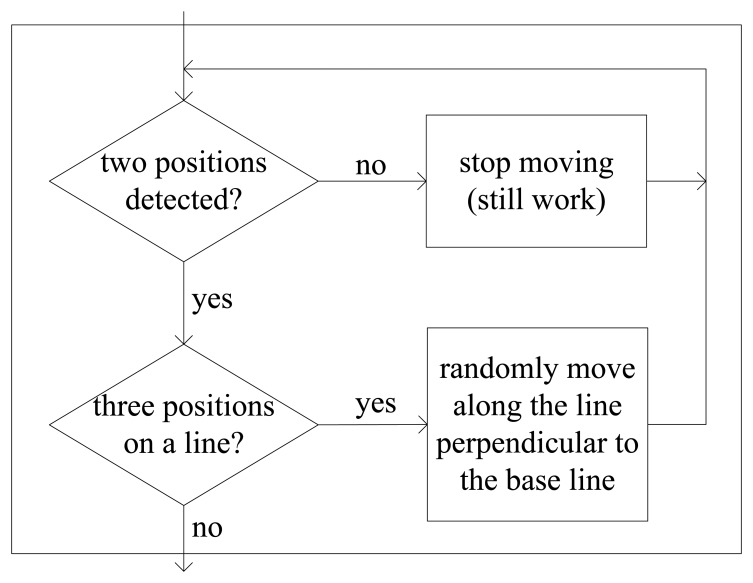
The detail of modified sub-algorithm in Figure 6.

Here, despite the fact the three robotic sensors locate in a line, any one is still at the detectable range of the other two. In Figure 7, the shadowed block is a blocked area for R_i1_ which is caused by the middle robotic sensor, R_i_, while R_i2_ precisely locates at the blocked area, as a result, R_i1_ isn't able to detect R_i2_. Similarly, R_i2_ isn't able to detect R_i1_ for the same reason. Therefore, we suggest a modifying sub-algorithm as illustrated in Figure 8 for robotic sensors in practice. During E formation configuration, once a robotic sensor detects only one neighbor, the robotic sensor should stop moving but still continue to detect and calculate processes. When a robotic sensor only detects two neighbors and further finds that it and its two neighbors are residing on a line, the robotic sensor can confirm that itself is in the middle. The robotic sensor will randomly choose one direction along the line which is perpendicular to the base line where the three robotic sensors locate and move towards that direction. Consequently, any one robotic sensor at the end will be able to detect the other at the end and begin to execute the CA or AA algorithms which are part of the TFA algorithm. Thus the deadlock situation of the three robotic sensors which locate on a line is broken.

## 4. Stability Analysis for TFA

The design procedure of TFA has been stated in the last section with the description of related pseudo codes. The theoretic results of the TFA used for three neighboring robotic sensors to configure an E formation are presented in detail in this section. The same TFA algorithm is executed on each robotic sensor independently and asynchronously. A global coordinate system is assumed for analysis.

*Lemma* 1: The average position *p̄* of three neighboring robotic sensors is invariable during clustering process, *i.e.*, $\dot{\bar{p}}=0$.

*Proof*: During clustering process, each robotic sensor executes the same CA algorithm, as shown in Figure 5a. Seen from CA algorithm principle, there is *p_ig_* = *p̄*, *i*∈ID, and considering the motion control equation Equation (2), we would have:

$$\begin{array}{c} {\dot{p}}_{i}=-\text{C}(p_{i}-p_{ig})=-\text{C}(p_{i}-\bar{p}),{\dot{p}}_{i1}=-\text{C}(p_{i1}-p_{i1g})=-\text{C}(p_{i1}-\bar{p}),\\ {\dot{p}}_{i2}=-\text{C}(p_{i2}-p_{i2g})=-\text{C}(p_{i2}-\bar{p}). \end{array}$$

Since $\bar{p}=\left(\frac{p_{i}+p_{i1}+p_{i2}}{3}\right)$, after derivative operation for two sides of this equation, then we can obtain:

$$\begin{array}{ll} \dot{\bar{p}} & =\left(\frac{{\dot{p}}_{i}+{\dot{p}}_{i1}+{\dot{p}}_{i2}}{3}\right)\left(\frac{-\text{C(}p_{i}-\bar{p})-\text{C(}p_{i1}-\bar{p})-\text{C(}p_{i2}-\bar{p})}{3}\right)=-\text{C}\left(\frac{p_{i}+p_{i1}+p_{i2}}{3}-\bar{p}\right).\\ & =-\text{C(}\bar{p}-\bar{p})=0 \end{array}$$

Lemma 1 has been proved.

*Lemma 2*: Three neighboring robotic sensors which execute basic CA algorithms can cluster into the neighborhood *B_η_*(*p̄*) with radius *η* around the center (*p̄* after spent time *_T_* :

$$B_{\eta }(\bar{p})=\left\{p|∥p-\bar{p}∥<\eta \right\},T=\frac{1}{\text{C}}\ln\left(\frac{M}{\eta }\right)$$

where *η* may be any positive number and *M* = max(‖*p_i_*(0)−*p̄:i*∈ID).

*Proof*: According to lemma 1, the average position of three neighboring robotic sensors executing the same CA algorithms satisfies *ṗ* = 0. For any robotic sensor R_i_, *i*∈ID, denote the error variable as *e_i_*(*t*) = *p_i_* − *p_ig_*, and the energy function as $V_{i}(t)=\frac{1}{2}e_{i}^{T}(t)e_{i}(t)$, after an operation of derivative is used for this function, we may have:

$$\begin{array}{ll} {\dot{V}}_{t}(t) & ={\left({\dot{e}}_{t}(t)\right)}^{T}e_{t}(t)={\left({\dot{p}}_{i}-{\dot{p}}_{ig}\right)}^{T}(p_{i}-p_{ig})\\ & \begin{array}{ll} ={\left({\dot{p}}_{i}-\dot{\bar{p}}\right)}^{T}(p_{i}-\bar{p}) & \left(\text{during clustering process}p_{ig}=\bar{p}\right) \end{array}\\ & ={\left({\dot{p}}_{i}\right)}^{T}(p_{i}-\bar{p})=-\text{C}{\left(p_{i}-\bar{p}\right)}^{T}(p_{i}-\bar{p})\\ & =-\text{C}{\left\|p_{i}-\bar{p}\right\|}^{2}\leq 0. \end{array}$$

To ∀ *η* > 0, when ‖ *e_i_*(*t*)‖ = ‖ *p_i_*−*p̄*‖> *η*, always *V̇_i_*(*t*) < 0. From Lyapunov stability theory, robotic sensor R_i_ can go into the neighborhood with η around the center p̄ after spending enough time *T_i_*.

Since *V̇_t_*(*t*)−C‖*p_i_*−*p̄*‖2 = −C(*e_i_*(*t*))T *e_i_*(*t*)= −2*CV_i_*(*t*), the solution of this differential equation is *V_i_*(*t*) = *V_i_*(0)*e*^−2^*^Ct^*. Here, when ‖*e_i_*(*t*)‖, R_i_ is considered to have gone into the η neighborhood of *p̄* and *T_i_* is used to denote the time spent on going into the neighborhood. From the energy function, there is $V_{i}(T_{i})=\frac{\eta^{2}}{2}$, and solving equation $V_{i}(0)e^{-2CT_{i}}=\frac{\eta^{2}}{2}$, the result is $T_{i}=\frac{1}{\text{C}}\ln\left(\frac{\sqrt{2V_{i}(0)}}{\eta }\right)$. Therefore, the shortest time which three neighboring robotic sensors have to spend to go into η neighborhood of *p̄* is:

$$\begin{array}{ll} T & =\max(T_{i}:i\in \text{ID})=\max\left(\frac{1}{\text{C}}\ln\left(\frac{\sqrt{2V_{i}(0)}}{\eta }\right):i\in \text{ID}\right)=\max\left(\frac{1}{\text{C}}\ln\left(\frac{\left\|p_{i}(0)-\bar{p}\right\|}{\eta }\right):i\in \text{ID}\right)\\ & =\frac{1}{\text{C}}\ln\left(\frac{M}{\eta }\right),\text{where}M=\max(∥p_{i}(0)-\bar{p}∥:i\in ID). \end{array}$$

Lemma 2 has been proved.

*Lemma 3*: Three neighboring robotic sensors can self-organize into AF from any CF successfully and in addition this is an irreversible process for the TFA algorithm.

*Proof*: According to Lemma 2, three neighboring robotic sensors which execute CA algorithms can cluster enough such that max(‖*p_i_* − *p_j_*‖:*I, j* ∈ ID,*i* ≠ *j*) ≤ η, let η = 2D then Equation (4) holds. Thus, the former part of Lemma 3 is proved.

**Figure 9.**
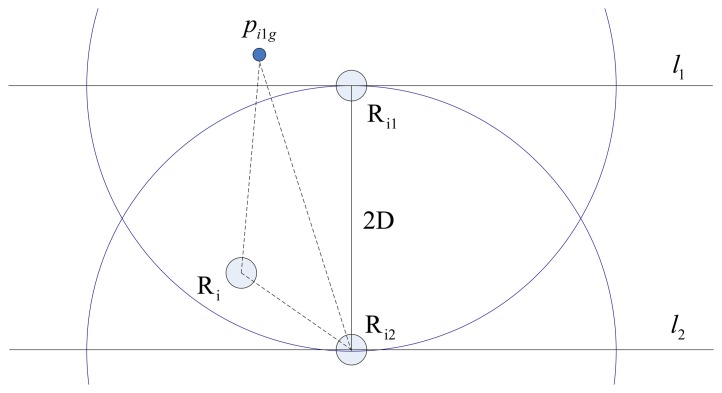
The critical formation necessary for CF transforming into AF.

If AF is transformed into CA, a critical formation as shown in Figure 9 must be experienced at this time step where max(‖*p_i_* − *p_j_*‖:*I, j* ∈ ID,*I* ≠ *j*) = 2D. Without the loss of generality, we only need to consider the case where robotic sensor R_i_ locates in the area formed by the two circles and the line formed by R_i1_R_i2_. It should be noted that the length of line segment R_i1_R_i2_ is equal to 2D and the circles shown are only used for analysis, they are not the maximum detectable boundaries of robotic sensors.

Obviously, if the transformation from AF into CF happens, deviation movement between R_i1_ and R_i2_ must first happen at this time step. Considering the symmetry, here we only discuss R_i1_, as a similar analysis is suitable for R_i2_ as well. During the deviation movement, the motion direction of R_i1_ must be upward line *l*_1_ The line *l*_1_ is vertical to line R_i1_R_i2_. That means the goal position *p_i_*_1_*_g_* of R_i1_ must be in the area above line *l*_1_. As a result, the distance from *p_i_*_1_*_g_* to R_i2_ must be larger than 2D. However, robotic sensor R_i1_ is still in AF and executing the AA algorithm of its adjusting process. Seen from the adjusting process, the distance from *p_i_*_1_*_g_* to R_i2_ must be less than 2D, which causes a contradiction, so the assumption of *p_i_*_1_*_g_* at the above of *l*_1_ doesn't hold. Similar analysis for R_i2_ can deduct that *p_i_*_2_*_g_* cannot be below *l*_2_. Therefore, the deviation movement between R_i1_ and R_i2_ can't happen since they are in AF. The latter part of Lemma 3 is proved.

Combining above, Lemma 3 has been proved.

*Lemma 4*: Three neighboring robotic sensors which execute basic AA algorithms can directly self-organize into an E formation starting from any AF.

*Proof:* For any robotic sensor R_i_, *i* ∈ ID, since the goal position is invariable during the interval between two time steps, so there is *ṗ_ig_* = 0. We define the error function as *e_i_*(*t*) = *p_i_* − *p_ig_* and the energy function as $V_{i}(t)=\frac{1}{2}{\left(e_{i}(t)\right)}^{T}e_{i}(t)$, then we would have:

$$\begin{array}{ll} V_{i}(t) & ={\left({\dot{e}}_{i}(t)\right)}^{T}e_{i}(t)={\left({\dot{p}}_{i}-{\dot{p}}_{ig}\right)}^{T}(p_{i}-p_{ig})={\left({\dot{p}}_{i}\right)}^{T}(p_{i}-p_{ig})=-\text{C}{\left(p_{i}-p_{ig}\right)}^{T}(p_{i}-p_{ig})\\ & =-\text{C}{\left\|p_{i}-p_{ig}\right\|}^{2}\leq 0 \end{array}$$

As seen from above, when ‖*p_i_−p_ig_*‖^2^>0, always *V̇_t_*(*t*) < 0. According to Lyapunov stability theory, *p_i_* asymptotically converges to *p_ig_*, *i.e.*, at last there is *p_i_* = *p_ig_*. Known from adjusting process definition, we have:

$$∥p_{ig}-p_{j}∥=\text{D},j\in NP_{i}$$

and after putting *p_i_* = *p_ig_* into the above equation, we can obtain:

$$∥p_{i}-p_{j}∥=\text{D},j\in NP_{i}.$$

That is to say, the distances of any robotic sensor R_i_ to its other two neighbors are equal to D. Therefore, the AF configured by the three robotic sensors finally transformed into an E formation. Lemma 4 has been proved.

*Theorem 1*: Three neighboring robotic sensors which execute the TFA algorithms can self-organize into an E formation starting from any initial distribution, *i.e.*, the TFA algorithm can solve Problem 1.

*Proof:* If the initial distribution of three neighboring robotic sensors is a CF formation, they will join the clustering process and execute CA algorithms. According to Lemma 3, the CF formation will transform into an AF formation and a reversible process never happens. According to Lemma 4 the AF formation will transform into an E formation. However, if the initial distribution of three neighboring robotic sensors is an AF formation, according to Lemma 4, the AF formation will directly transform into an E formation. The combination of the above indicates that the three neighboring robotic sensors can self-organize into an E formation regardless of their initial distribution. The theorem has thus been proved.

## 5. Simulation Studies

In the last two sections, we first designed the interactive control algorithm, TFA, and then stated the related theoretic results which indicate that three neighboring robotic sensors can self-organize into an E formation regardless of their initial distribution. In this section we aim to conduct simulation experiments on a computer so as to test the effectiveness of the TFA. All simulation programs were written in Steve based on the Breve platform [27]. Breve is a 3D simulation environment for the simulation of decentralized systems and artificial life, using the object oriented language, Steve, as programming language. Breve also contains a commonly used class library which assists with fast model simulation and algorithm test. The interested reader is referred to our earlier work [28] for the detailed simulation programs in Steve used in this section.

Parameter settings for simulation experiments are as following: side-length of the desired E formation, D = 10 (units); detection radius of a robotic sensor, r_s_ = 50 (units); maximal speed of robotic sensor, ‖*v*_max_‖=10 (units/s;; control constant of Equation (2), C=‖*v*_max_‖ /r*_s_* = 0.2, which satisfies Equation (3); at the beginning of simulations, robotic sensors are randomly distributed in the circle area whose radius is r_s_ /2, which ensures any one robotic sensor can detect all the other ones; robot sensor R_i_ is instructed to stop running when ‖*v_i_*‖<0.01 where ‖*v_i_*‖ means instant speed of R_i_, in other words, stop condition is specified for the simulation of E formation behavior. In fact, the limited ‖*v_i_*‖ of R_i_ reflects the deviation degree between *p_i_* and *p_ig_*, which can be seen from Equation (2). To be convenient to evaluate the change of the close degree between the current formation and the desired E formation over time, we defined average side-length error, E_asl_(*t*), as formula:

(8) $${\text{E}}_{\text{as}1}(t)=\frac{\sum_{i,j\in \text{ID}\wedge i\neq j}\left|∥p_{i}(t)-p_{j}(t)∥-\text{D}\right|}{\text{card}(\text{ID})}$$

in Equation (8), *card*(ID) represents the number of elements in set ID.

**Figure 10.**
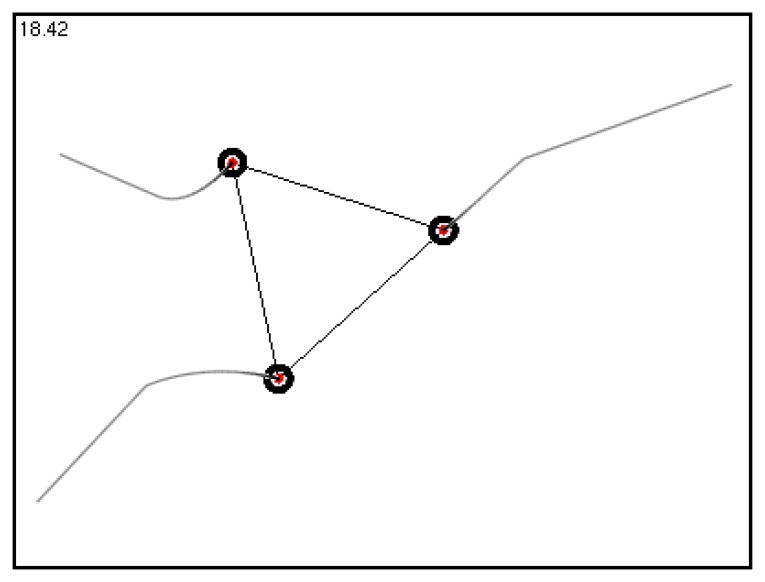
The E formation behavior of three robotic sensors starting from CF-2 distribution.

We simulated an E formation behavior for three robotic sensors extensively based on typical initial distributions. These distributions are: (1) CF-1 (17.47, 16.06, 30.42), elements are side-length values and only one side-length value is larger than 2D; (2) CF-2 (36.66, 15.75, 30.56), two side-length values are larger than 2D; (3) CF-3 (21.94, 24.49, 27.00), all three side-length values are larger than 2D; (4) AF (11.60, 15.69, 11.61), all three side-length values are less than 2D, *i.e.*, Equation (4) holds. The simulations of E formation behavior demonstrate that three neighboring robotic sensors can self-organize into an E formation starting from these typical initial distributions using TFA. For instance, Figure 10 shows the trajectories of three robotic sensors during an E formation configuration starting from distribution CF-2.

**Figure 11.**
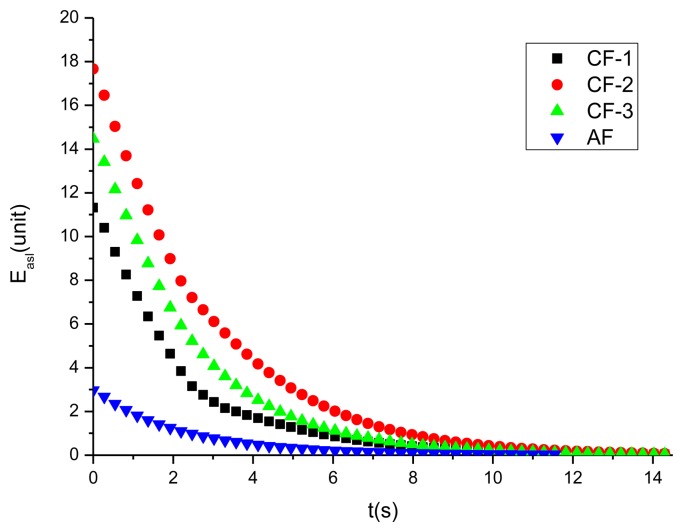
The changes of average side-length error over time under typical initial distributions.

The changes of average side-length errors over the time under these typical distributions are illustrated in Figure 11. The error curves indicate that those typical initial formations configured by three robotic sensors can always transform into the desired E formation with side-length D. In short, since these typical distributions summarize all the possible initial distributions, computer simulations show us that three neighboring sensors can self-organize into an E formation through executing the same TFA algorithm starting from any initial distribution.

## 6. Conclusions and Future Work

In this paper, first, we established the state transition model for real robotic sensors. Then, the design procedure of the TFA algorithm is presented in detail. The same TFA algorithm is executed by each robotic sensor independently and asynchronously during E formation configuration. Finally, we conducted stability analysis and simulation verification for the TFA algorithm. Theoretical analysis and computation simulations both indicate that the proposed TFA algorithm can ensure that three neighboring robotic sensors self-organize into an E formation regardless of their initial distributions. However, the TFA algorithm is not our ultimate goal. In our future work, we are interested in extending it to controlling large scale robotic sensors to configure a uniform sensor network which has a specific local formation.

## Figures and Tables

**Table 1.** The pseudo code of the basic AA algorithm.

Algorithm 1 for Adjusting Process
FUNCTION *p_ig_* = *φ_AA_*(*NP_i_*)
R_i_ calculates its goal position *p_ig_* which satisfies Equations (5) and (6) according to Figure 3b–e.

**Table 2.** The pseudo code of basic CA algorithm.

Algorithm 2 for Clustering Process
FUNCTION *p_ig_* = *φ_CA_*(*NP_i_*)
$\bar{p}=\frac{p_{i}+p_{i1}+p_{i2}}{3}$; //*pi* = (0,0) is the origin of R_i_'s local coordinate system.
*p_ig_* = *p̄*;

**Table 3.** The pseudo code of TFA algorithm.

Algorithm 3 used for E formation behavior
FUNCTION *p_ig_* = *φ_TFS_* (*NP_i_*)
IF G is CF THEN
*p_ig_* = *φ_CA_*(*NP_i_*);
ELSE *p_ig_* = *φ_AA_* (*NP_i_*);
END IF

## Notes

**Author Contributions:** All authors contributed equally to this work. Xiang Li designed the interactive control algorithm and performed theoretic analysis. Hongcai Chen supervised the project and assisted with experiments. Xiang Li wrote the paper. All authors discussed the results and implications and commented on the manuscript at all stages.
